# Histopathologic predictors of recurrence and survival in early T stage oral tongue squamous cell carcinoma

**DOI:** 10.3389/froh.2024.1426709

**Published:** 2024-08-06

**Authors:** Benjamin J. Damazo, Nihal A. Punjabi, Yuan F. Liu, Jared C. Inman

**Affiliations:** ^1^ Department of Otolaryngology-Head and Neck Surgery, Loma Linda University Medical Center, Loma Linda, CA, United States; ^2^ Case Western Reserve University School of Medicine, Cleveland, OH, United States

**Keywords:** head and neck, recurrence, squamous cell carcinoma, radiotherapy, cancer

## Abstract

**Objectives:**

Recurrence and survival in early T-stage oral tongue squamous cell carcinoma (OTSCC) may be impacted by histopathologic risk factors. This study aims to examine which of these factors predict long-term outcomes of T1 and T2 OTSCC.

**Methods:**

A retrospective review of T1 and T2 OTSCC patients treated with surgery at a single tertiary care center was conducted. Multivariate regression and Kaplan-Meier survival plots were used to identify predictors of recurrence and compare disease-free survival respectively.

**Results:**

100 consecutive patients were studied. Of these, 51 were staged pT1, 49 pT2, 69 pN0, 10 pN1, and 21 pN2. Multivariate regression analysis revealed that >4 nodes was the strongest predictor of overall recurrence [odds ratio 1.68 (1.23–2.28), *p* = 0.001], while >4 nodes [odds ratio 1.14 (1.09–1.85), *p* = 0.008] and pT2 [odds ratio 1.15 (1.01–1.30), *p* = 0.033] were predictors of local recurrence (R2 = 0.112). Five-year disease-free survival was not significantly impacted by any risk factors except for the number of positive nodes—86% for ≤4 nodes vs. 20% for >4 nodes (*p* < 0.001)—and pathologic T-stage—90% for pT1 vs. 75% for pT2 (*p* = 0.035) regardless of adjuvant radiation and/or chemotherapy use.

**Conclusions:**

Patients who underwent adjuvant radiation and/or chemotherapy had similar survival to those who did not despite having worse overall tumor prognostic factors. Adding adjuvant therapy may equalize some high-risk histopathologic factors. In the highest risk patients—specifically those with pathologic >4 nodes and pT2 staging—adjuvant therapy should be considered.

## 1 Introduction

Oral cavity cancer is among the most prevalent head and neck malignancies, with oral tongue squamous cell carcinoma (OTSCC) being the most common variant (1). Typically, OTSCC is treated with primary surgery followed by adjuvant radiation and/or chemotherapy depending on the disease stage and tumor histopathologic features. Regional cervical metastasis rates vary from 15% to 47% with occult rates reaching up to 42% (2–5). Achieving disease cure requires complete surgical resection and appropriate adjuvant treatment, selected based on histopathologic risk stratification. Some histologic tumor features have been shown to predict survival and recurrence, such as perineural invasion (PNI) and tumor budding (6, 7). However, uncertainty remains regarding the value of other tumor characteristics (e.g., lymphovascular invasion [LVI], tumor differentiation, extracapsular spread [ECS], and number of positive nodes) as risk factors for recurrence and prognosticators for survival (8, 9). Understanding how these histopathologic risk factors predict long-term outcomes is essential to identify patients that would benefit from adjuvant therapy. This is especially important in early T stage (T1 and T2) OTSCC, where primary tumor resection is achievable and need for additional treatment may be unclear. We examined long-term outcomes in a cohort of patients with T1 and T2 OTSCC treated with primary surgical resection, elective or therapeutic neck dissection, and adjuvant radiation and/or chemotherapy. We aimed to identify predictors of local and regional recurrence and examine differences in survival based on these predictors.

## 2 Methods

This study received ethical approval from Loma Linda University Medical Center IRB (Approval #5150330). This is an IRB-approved retrospective study, all patient information was deidentified and patient consent was not required. We performed a review of a cohort of newly diagnosed and previously untreated adult patients with T1 and T2 OTSCC treated at LLUMC over a twelve-year consecutive period with a minimum five-year follow-up. Patients were identified through a query of the institutional electronic medical records database for all oral tongue cancers by International Classification of Diseases, Ninth Revision (ICD-9) codes and through a query of the institutional tumor registry. Patients with non-squamous histology, previous treatment for any head and neck cancer, and incomplete medical records were excluded. All staging was based on the American Joint Committee on Cancer (AJCC) 7th edition using surgical pathology findings based on glossectomy and neck dissection. Pathologic findings were reviewed by a head and neck trained pathologist. Disease free survival (DFS)—the length of time after treatment that the patient survives without any signs or symptoms of recurrence—and overall survival—the length of time after treatment that the patient is still alive—were both recorded.

### 2.1 Statistics

The Student *t*-test and the Fisher exact test were used to compare continuous and categorical variables, respectively. A univariate Cox proportional hazards regression model was used to find predictors of overall, local, regional and distal recurrence. A multivariate Cox proportional hazards regression was performed in a step-forward manner to find predictors of recurrence, considering interactions between variables. Survival curves were constructed using the Kaplan-Meier method for significant predictors of recurrence. The log-rank test was used to compare survival curves. Means are reported as mean ± standard deviation (SD). Significance was established at the *p* < 0.05 level. Hazards ratios (HR) with 95% confidence intervals (CI) are reported as HR [lower 95% CI, upper 95% CI]. Adjusted correlation coefficients (R2) are reported for significant variables in regression analyses. All statistical analyses were preformed using SPSS (SPSS Inc., Chicago, IL).

## 3 Results

### 3.1 Demographics and tumor characteristics

One hundred patients were included: 41 women and 59 men with a mean age of 61.7 ± 15.7 years (range 19–98 years). All patients underwent primary surgical therapy consisting of partial glossectomy and neck dissection. The mean follow-up time was 61.7 ± 49.7 months (range 1–261 months, median 45.8 months). Sixty-two patients were smokers and 59 were alcohol consumers. Fifty-one patients had pT1 disease, while 49 had pT2 disease. Sixty-nine were staged pN0, 10 patients were pN1, and 21 were pN2.

Overall, 18 patients developed recurrence, (12 local, 8 regional, 5 distant). Significantly fewer recurrences occurred in pT1 than pT2 patients (10% vs. 27%, *p* = 0.038), in pN0 than pN + patients (12% vs. 32%; *p* = 0.021), and in those without ECS (15% vs. 50%, *p* = 0.033). Of the pN + patients, those with ≤4 metastatic nodes developed significantly fewer recurrence (15%) vs. patients with >4 metastatic nodes (67%, *p* = 0.009). A summary of demographics and tumor characteristics is presented in Table 1.

**Table 1:** Demographics and tumor variables by recurrence.

	Mean ± SD or number (% within group)			*p*
	All (*n* = 100)	Recurrence (*n* = 18)	No recurrence (*n* = 82)	
Age (years)	61.7 ± 15.7	63.8 ± 11.2	61.2 ± 16.5	0.530
Male	59	11 (61)	48 (59)	1.000
Female	41	7 (39)	34 (41)	
Smoking	62	13 (72)	49 (60)	0.425
Alcohol	59	7 (58) (*n* = 12)	38 (59) (*n* = 64)	1.000
Death	48	14 (78)	34 (41)	*0.008
F/u time (months)	61.7 ± 49.7	63.5 ± 68.7	60.5 ± 45.0	0.817
pT1	51	5 (28)	46 (56)	*0.038
pT2	49	13 (72)	36 (44)	
pN0	69	8 (44)	61 (74)	*0.021
pN1	10	2 (11)	8 (10)	
pN2	21	8 (44)	13 (16)	
Margin +	7	2 (11)	5 (6)	0.606
PNI	21	2 (11)	19 (23)	0.348
LVI	12	3 (17)	9 (11)	0.448
Poor diff	24	5 (28)	19 (23)	0.762
ECS	8	4 (22)	4 (5)	*0.033
>4 nodes	6	4 (22)	2 (2)	*0.009
Radiation	33	8 (44)	25 (30)	0.277
Chemotherapy	8	1 (6)	7 (9)	1.000
Local recurrence	12	12 (67)	0 (0)	n/a
Neck recurrence	8	8 (44)	0 (0)	
Metastasis	5	5 (28)	0 (0)	

*P*-values refer to comparison between recurrence and disease-free groups. SD, standard deviation; F/u, follow-up; PNI, perineural invasion; LVI, lymphovascular invasion; Poor diff, poorly differentiated; ECS, extracapsular spread.

Significant results are indicated by asterisks.

### 3.2 Patients receiving radiation therapy and chemotherapy

Of the 100 patients, 33 received adjuvant radiation therapy (RT) and 8 received both adjuvant RT and chemotherapy. Demographics and tumor characteristics of patients by adjuvant therapy are presented in Table 2.

**Table 2:** Demographics and tumor variables by treatment type.

	Mean ± SD or number (% within group)		*p*	Mean ± SD or number (% within group)		*p*
	RT (*n* = 33)	No RT (*n* = 67)		Chemo (*n* = 8)	No chemo (*n* = 92)	
Age (years)	59.1 ± 15.8	62.9 ± 15.6	0.258	49.5 ± 21.3	62.7 ± 14.8	*0.022
Male	24 (73)	35 (52)	0.056	6 (75)	53 (58)	0.466
Female	9 (27)	32 (48)		2 (25)	39 (42)	
Smoking	19 (58)	43 (64)	0.662	4 (50)	58 (63)	0.474
Alcohol	15 (63) (*n* = 24)	30 (58) (*n* = 52)	0.467	4 (80) (*n* = 5)	41 (58) (*n* = 71)	0.643
Death	15 (45)	37 (55)	0.400	4 (50)	48 (52)	1.000
F/u time (months)	45.5 ± 38.9	68.7 ± 52.8	*0.028	32.8 ± 23.2	63.5 ± 50.6	0.094
pT1	13 (39)	38 (57)	0.137	1 (13)	50 (54)	*0.029
pT2	20 (61)	29 (43)		7 (87)	42 (46)	
pN0	17 (52)	52 (78)	*0.011	1 (13)	68 (74)	*0.001
pN1 or pN2	16 (48)	15 (22)		7 (87)	24 (26)	
Margin +	5 (13)	2 (3)	*0.038	2 (25)	5 (5)	0.096
PNI	13 (39)	8 (12)	*0.003	3 (38)	18 (20)	0.359
LVI	5 (15)	7 (10)	0.523	3 (38)	9 (10)	*0.031
Poor diff	12 (36)	12 (18)	*0.050	2 (25)	22 (33)	1.000
ECS	5 (15)	3 (4)	0.111	3 (38)	5 (5)	*0.016
>4 nodes	3 (9)	3 (4)	0.393	2 (25)	4 (4)	0.072
Overall recurrence	8 (24)	10 (15)	0.277	1 (13)	17 (18)	1.000
Local recurrence	5 (15)	7 (10)	0.524	0 (0)	12 (13)	0.591
Neck recurrence or mets	4 (12)	4 (6)	0.433	1 (13)	10 (11)	1.000

*P*-values refer to comparison between treatment and no treatment for RT or chemotherapy. SD, standard deviation; RT, radiation therapy; F/u, follow-up; PNI, perineural invasion; LVI, lymphovascular invasion; Poor diff, poorly differentiated; ECS, extracapsular spread; mets, metastasis.

Significant results are indicated by asterisks.

Patients with generally worse tumor characteristics were more likely to receive RT. These characteristics included significantly more pN + disease (*p* = 0.011), positive margins (*p* = 0.038), PNI (*p* = 0.003), and poorly differentiated cancer (*p* = 0.050). Similarly, patients who received chemotherapy had significantly more pT2 disease (*p* = 0.029), pN + disease (*p* = 0.001), LVI (*p* = 0.031), and ECS (*p* = 0.016).

Local, regional, and overall recurrence rates were similar when patients with poor histopathologic factors were selected to receive post-operative RT and chemotherapy, except in the groupings of pT2, ECS, pN+, and >4 LN + . In these groups, despite RT or chemotherapy, recurrence rates were still higher than their comparative group.

### 3.3 Recurrence

Univariate Cox regression showed that pT2, pN+, ECS, and >4 nodes were significant predictors of overall recurrence. For local recurrence, pT2, ECS, and >4 nodes were significant predictors. There were no significant predictors of neck recurrence or distant metastasis. *P*-values and hazards ratios for predictors of recurrence are presented in Table 3.

**Table 3:** Predictors of recurrence with univariate Cox regression.

	Recurrence		Local recurrence		Neck recurrence or mets	
	*p*	HR (95% CI)	*p*	HR (95% CI)	*p*	HR (95% CI)
Age (years)	0.53	1.00 (0.99, 1.01)	0.584	1.00 (0.99, 1.01)	0.63	1.00 (0.99, 1.00)
Male	0.843	1.01 (0.87, 1.19)	0.569	1.04 (0.91, 1.19)	0.75	0.98 (0.86, 1.11)
Female		0.98 (0.84, 1.15)		0.96 (0.84, 1.10)		1.02 (0.90, 1.16)
Smoking	0.329	1.08 (0.92, 1.27)	0.328	1.07 (0.93, 1.22)	0.91	1.01 (0.89, 1.15)
Alcohol	0.947	0.99 (0.84, 1.18)	0.638	0.97 (0.85, 1.10)	0.36	0.94 (0.82, 1.08)
pT1	*0.030	0.85 (0.73, 0.98)	*0.011	0.85 (0.75, 0.96)	0.7	0.96 (0.86, 1.11)
pT2		1.18 (1.02, 1.37)		1.18 (1.04, 1.34)		1.02 (0.90, 1.16)
pN0	*0.013	0.81 (0.69, 0.96)	0.132	0.90 (0.78, 1.03)	0.08	0.89 (0.78, 1.01)
pN1		1.23 (1.05, 1.44)		1.11 (0.97, 1.28)		1.13 (0.99, 1.29)
pN2						
Margin +	0.455	1.12 (0.83, 1.51)	0.849	1.02 (0.79, 1.32)	0.78	1.04 (0.81, 1.32)
PNI	0.26	0.90 (0.74, 1.08)	0.698	0.97 (0.83, 1.14)	0.07	0.87 (0.75, 1.01)
LVI	0.506	1.08 (0.85, 1.37)	0.6	1.05 (0.86, 1.29)	0.51	1.07 (0.88, 1.29)
Poor diff	0.682	1.04 (0.87, 1.24)	0.932	1.01 (0.86, 1.17)	0.79	1.02 (0.88, 1.18)
ECS	*0.014	1.42 (1.08, 1.86)	*0.021	1.32 (1.04, 1.67)	0.19	1.16 (0.93, 1.46)
>4 nodes	*0.001	1.68 (1.23, 2.28)	*0.003	1.50 (1.15, 1.95)	0.07	1.27 (0.98, 1.64)
Radiation	0.259	1.10 (0.93, 1.29)	0.501	1.05 (0.91, 1.20)	0.291	1.06 (0.95, 1.19)
Chemotherapy	0.677	0.94 (0.71, 1.25)	0.281	0.88 (0.69, 1.11)	0.629	1.05 (0.86, 1.28)

Mets, metastasis; HR, hazards ratio; CI, confidence interval; PNI, perineural invasion; LVI, lymphovascular invasion; Poor diff, poorly differentiated; ECS, extracapsular spread.

Significant results are indicated by asterisks.

Multivariate Cox regression showed that >4 nodes was the only significant predictor of overall recurrence [HR 1.68 (1.23–2.28), *p* = 0.001]. The presence of >4 nodes [HR 1.14 (1.09, 1.85), *p* = 0.008] and pT2 [HR 1.15 (1.01, 1.30), *p* = 0.033] were the only significant predictors of local recurrence. Again, there were no significant predictors of neck recurrence or distant metastasis.

### 3.4 Survival

**Figure 1:**
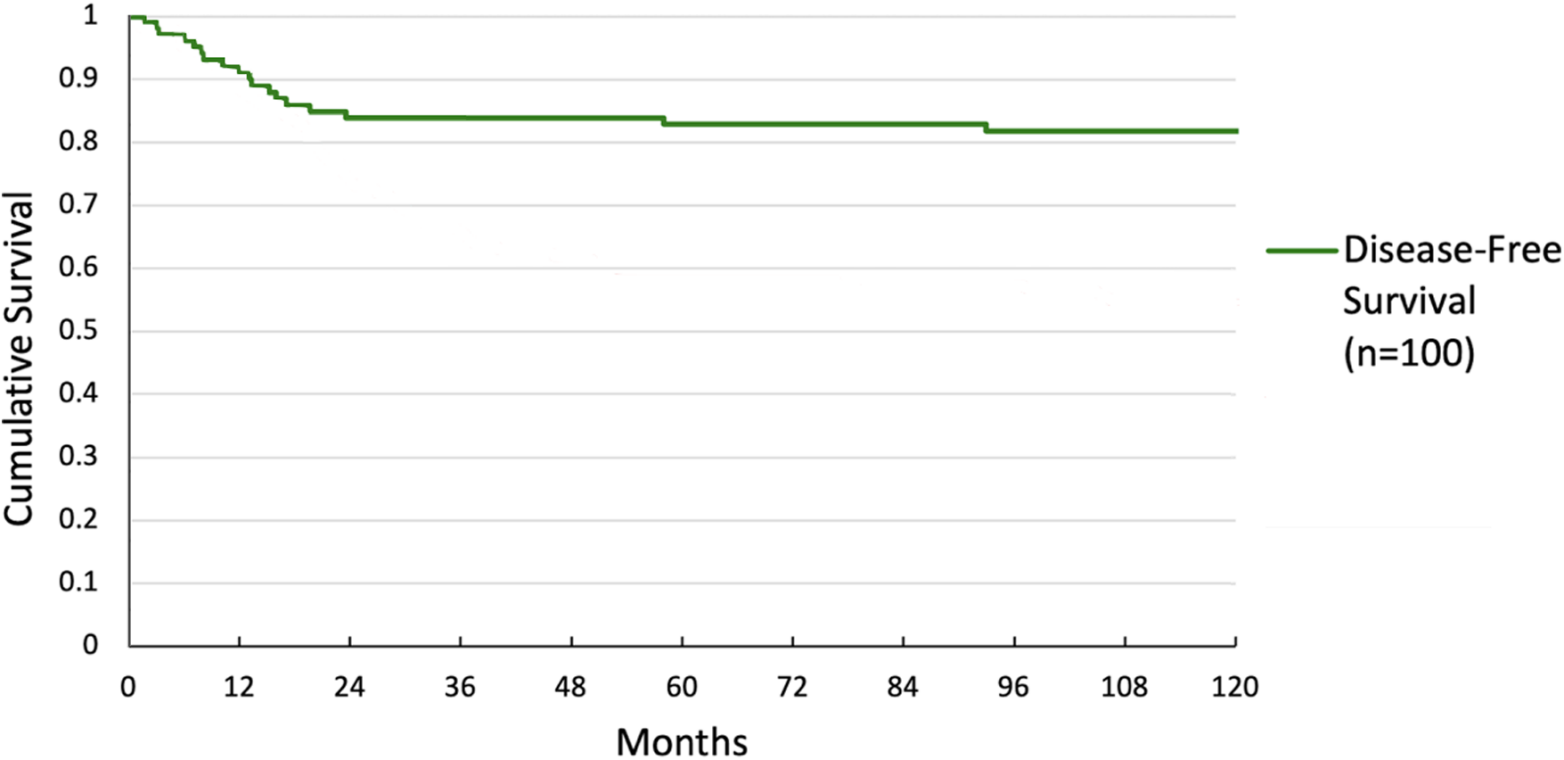
Disease-free survival in all patients.

**Figure 2:**
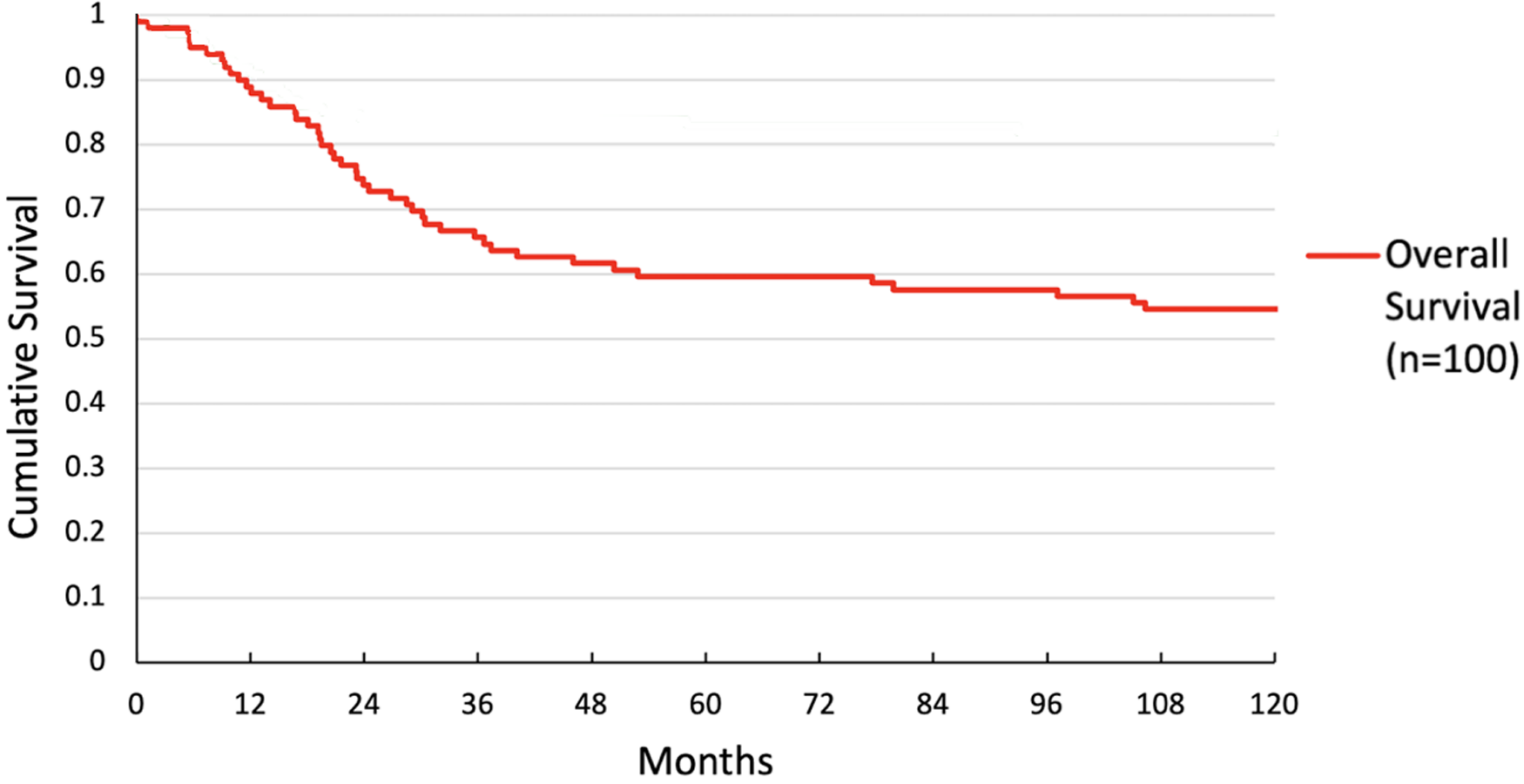
Overall survival in all patients.

Overall survival was 59.6% at 5 years and 54.5% at 10 years. DFS was 82.8% at 5 years and 81.8% at 10 years. Overall and DFS are shown in Figures 1, 2.

**Figure 3:**
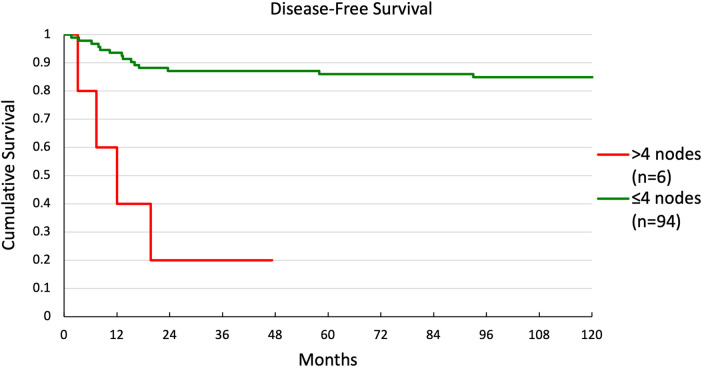
Disease-free survival in patients with >4 nodes or ≤4 nodes.

**Figure 4:**
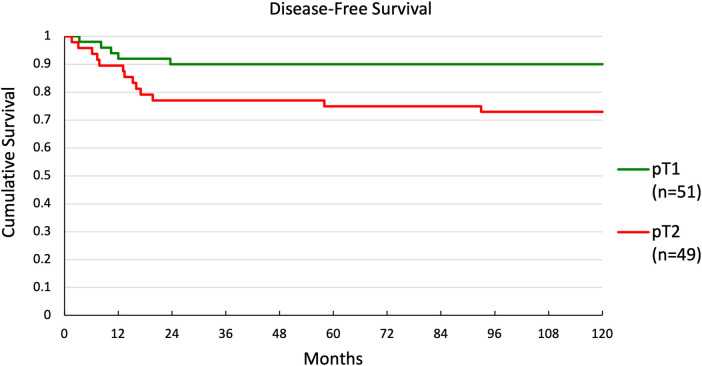
Disease-free survival in patients with pT1 or pT2 disease.

Multivariate and univariate Cox regression showed significant differences in DFS for two groups. Kaplan-Meier curves were generated for these groupings. DFS for patients with ≤4 nodes or >4 nodes is shown in Figure 3. There was a significant difference in survival between the 2 groups (*p* < 0.001), with 5-year survival at 86% and 20%, respectively. DFS for patients with pT1 or pT2 disease is shown in Figure 4. Again, there was a significant difference in survival between the 2 groups (*p* = 0.037), with 5-year survival at 90% and 75%, respectively.

## 4 Discussion

The incidence of oral tongue cancers has been increasing in the United States (10–13). Treatment typically consists of primary surgery followed by adjuvant radiation or chemotherapy (14). Early T stage OTSCC generally has worse overall survival compared to other early T stage head and neck cancers (15). These tumors may be difficult to cure due to uncertainty about which histopathologic features are relevant for risk stratifying patients and predicting recurrence (9, 16–19). Patients experience treatment failure when they develop metastasis, tumor recurrence, or disease persistence despite completing treatment (20). Accurately identifying aggressive tumors and appropriately selecting adjuvant therapy decreases treatment failures (21, 22).

In the literature, recurrence free survival for early T stage OTSCC ranges from 75% to 89% (22–25). In this study, the recurrence rates were similar, ranging from 75% to 90% disease free survival at 5-year follow-up. At 10-year follow-up DFS was maintained at 90% for patients with T1 disease but decreased to 72.9% in patients with T2 disease. To predict which patients with low stage disease should undergo adjuvant therapy, researchers have attempted to identify risk factors for recurrence (6, 7, 26, 27). Suggested tumor features such as ECS, nodal metastasis, tumor size, PNI, and LVI have been associated with recurrence (7, 24, 26, 28–37).

In contrast to prior studies, we found no association between recurrence and margin positivity, PNI, LVI, or poor differentiation (6, 38–41). This difference is likely due to confounding from the pattern of adjuvant treatment use in our cohort. Patients with high-risk features, including positive margins, PNI, LVI, and poor differentiation, were more likely to receive adjuvant therapy (Table 2) causing the appearance that these factors were not associated with disease recurrence. Despite possessing more high-risk features on average, patients who received adjuvant therapy had similar survival to those who did not, suggesting that multimodal treatments were able to minimize the adverse effects of these features.

Increased T stage, increased N stage, presence of >4 metastatic nodes, and ECS at the time of treatment were significantly associated with a higher risk for local recurrence by univariate regression (Table 3). Our Kaplan-Meier curves also showed significantly worse survival for T2 vs. T1–consistent with prior reports showing 5-year overall survival of 73% for T1% and 64% for T2–and for patients with >4 nodes vs. ≤4 nodes (42). Patients with ECS and T2 were more likely to receive chemotherapy than patients without these features, but they did not receive radiation at higher rates. Furthermore, the absolute number of patients receiving chemotherapy was low in both groups (3/8 for ECS and 7/49 for T2). Previous work has shown that radiation therapy can improve outcomes in patients with ECS (9). Since both these features were predictors of recurrence and T2 was also a predictor for survival, these groups may have benefitted from more aggressive adjuvant therapy. Similarly, while patients with higher N stage were more likely than those with N0 to receive radiation and chemotherapy, the absolute number treated was likely not high enough to significantly reduce recurrence in this group. Finally, patients with >4 positive nodes, which was the only feature predictive of recurrence on both univariate and multivariate analysis, were not more likely to receive either adjuvant therapy. While features such positive margins, PNI, and ECS are commonly considered when selecting patients for adjuvant therapy, our results suggest that the number of positive lymph nodes may be overlooked (6, 38–41). Given the strength of this feature to predict recurrence and survival, the presence of multiple positive lymph nodes alone may be important to consider when selecting patients for adjuvant therapy.

This study is limited by its retrospective nature and confounding from the use of adjuvant radiation and chemotherapy in patients with high-risk histopathologic features, possibly masking the effects of these features. Our long follow-up time also made it unfeasible to utilize the most up-to-date staging system. In the AJCC 8th edition staging system, depth of invasion (DOI) is now included in the T classification. Therefore, it is possibly that some of the tumors we assessed may have been upstaged to T3 or T4 using the 8th edition. The DOI data for the earliest patients in this cohort was not detailed enough to restage them based on the 8th edition, so the 7th edition was used to ensure internal validity. Despite these limitations, this study provides long-term outcomes for patients with T1 and T2 OTSCC and helps identify high-risk histopathologic features to guide adjuvant treatment plans. In many studies data for follow-up stops after 5 years, but this study presents data up to10 years following diagnosis. Future large, multi-institutional, randomized controlled trials would further elucidate the impact of certain risk factors on recurrence and survival, increasing clinicians’ ability to risk stratify patients.

## 5 Conclusions

Even in early T stage OTSCC, post-operative adjuvant treatment in patients with high-risk histopathologic features can allow for equivocal survival when compared to lower risk patients. Our study identified pN1 and pN2, pT2, ECS, and multiple nodes as univariate risk factors for recurrence and multiple nodes as the only significant risk factor on multivariate analysis. This suggests that these factors should be considered when selecting patients for adjuvant treatment. Furthermore, the number of involved nodes may serve as an independent indicator for adjuvant treatment.

## Data Availability

The datasets presented in this article are not readily available because the IRB protocols at our institution restrict sharing of patient data. Requests to access the datasets should be directed to jinman@llu.edu.
